# PROTOCOL: The effectiveness of social protection interventions in low‐ and middle‐income countries: An evidence and gap map

**DOI:** 10.1002/cl2.1160

**Published:** 2021-07-07

**Authors:** Latha Thimmappa, Ashrita Saran, Sonia R. B. D'Souza, Binil V.

**Affiliations:** ^1^ Manipal College of Nursing Manipal Manipal Academy of Higher Education Manipal India; ^2^ Director, Campbell South Asia New Delhi India

## Abstract

This evidence and gap map will provide an overview of the existing systematic reviews and impact evaluations on the key outcome domains and interventions aimed at improving social protection among people living in low‐ and middle‐income countries (LMICs). The specific objectives of this map are to: (1) Develop a clear framework of types of interventions and outcomes related to the effectiveness of interventions on social protection for people in LMICs. (2) Map available systematic reviews and primary studies on the effectiveness of interventions on social protection for people in LMICs. (3) Provide a structured and accessible collection of existing evidence and identifying gaps in the available evidence on social protection intervention, thereby helping to inform the research agendas of funders and other organisations.

## 1 BACKGROUND

### 1.1 Introduction

#### 1.1.1 The problem, condition or issue

The existence of social protection can be recognised as one of the most significant social achievements of the 20th century. Systems of social protection enable the societies to enhance the well‐being and security of their citizens by protecting them from vulnerability and deprivation so that they can pursue a decent life. Social protection can meet the essential needs of human survival by ensuring a basic social and economic security (García & Gruat, 2003).

Asian development bank defines social protection as a set of policies and programmes designed to reduce poverty and vulnerability by promoting efficient labour markets, diminishing people's exposure to risks, and enhancing their capacity to protect themselves against hazards and interruption/loss of income (Rawat, 2012). It is concerned with protecting and helping those who are poor and vulnerable, such as children, women, older people and people living with disabilities, the displaced, the unemployed and the sick. The social protection overlaps with a number of livelihoods, human capital and food security interventions (Harvey et al., 2007). Social protection is about people and families having security in the face of vulnerabilities, contingencies, having access to health care, and safety in working conditions. The goals of social protection interventions vary widely, from reducing poverty and vulnerability, building human capital, empowering women and girls, improving livelihoods, and responding to economic and other shocks.

The International Labour Organization (ILO) recommendation 202 recommends that “social protection floors” should have the minimum social security requirements such as essential healthcare services, all‐round development of children, unemployment, maternity care and disability security when the active age group is unable to earn and basic income security for older people (ILO, 2012). Social protection floors are a set of national social security guarantees that provide access to essential healthcare and basic income security for all who need it (ILO, 2012). Social protection systems help individuals and families, especially the poor and vulnerable, cope with crises and shocks, find jobs, improve productivity, invest in the health and education of their children, and protect the aging population.

Social protection is broader than social security which includes social services, protection against financial insecurity and social welfare. Social protection is designed to accommodate the assistance for catastrophes of life in partnership with the public and private sectors, signifying better outcomes. Its purpose is to increase the capacity to meet the needs of the people and promote human welfare (Mpedi, 2011).

Many countries have not improved their investment towards social protection. It is possible to gradually build social protection and, relying on comprehensive long‐term national social protection action plans. These action plans are expanding the social insurance schemes now in operation; building community or employment‐based insurance schemes on a voluntary basis, introducing and extending social welfare services, employment guarantee schemes and public finance and non‐contributory cash transfers (African union, 2008).

The map will cover a broad range of intervention for social protection among children, adults and geriatric populations focusing on low‐ and middle‐income countries (LMICs).

LMICs are defined by World Bank as low‐income economies—those with a Gross National Income (GNI) <$995; lower middle‐income economies—those with a GNI per capita between $996 and $3895; and upper middle‐income economies—those with a GNI per capita between $3896 and $12,055 (The World Bank, 2018). People living in LMICs experience many forms of insecurity and is a hard truth for the poorest of the poor in the informal economy. They are most in need of support and protection, yet they are the ones who are least protected, since we are far from realising that social protection is a right for all (García & Gruat, 2003).

The World Bank estimates that more than 1 billion people in developing countries participate in at least one social assistance programme (Gentilini et al., 2014). The ILO estimates that only 27% of the world's population has access to comprehensive social security systems (ILO, 2014). There is wide variation in coverage, with most programmes only reaching the middle poor and middle‐income countries, not the extreme poor (Gentilini et al., 2014). In recent years, the success of social protection interventions in middle income countries (MICs) such as Brazil and Mexico, along with the series of food, fuel, and financial crises, has prompted policymakers in low income countries and fragile situations to examine the possibility of introducing such programmes in their own countries (Andrews et al., 2012).

It is also essential to note that if social protection can improve the sustainable development goals (SDGs) of WHO, then the key global objectives should be poverty reduction and sustainable development. The World Bank Group supports universal access to social protection, and is central to its goals of ending poverty and boosting shared prosperity. Universal social protection coverage includes: providing social assistance through cash transfers to those who need them, especially children; benefits and support for people of working age in case of maternity, disability, work injury or for those without jobs; and pension coverage for the elderly. Assistance is provided through social insurance, tax‐funded social benefits, social assistance services, public works programmes and other schemes guaranteeing basic income security (The World Bank, 2019).

Although social protection can be a successful tool to lift people out of poverty, a special focus on the poorest of the households is necessary to increase their participation and benefits from the interventions (Kestere Van Kesteren et al., 2018). Also, to accelerate progress on social protection, it requires governments and development partners to have the best available data on what works best and where the gaps are in implementing social protection measures, it becomes essential to have an interactive evidence and gap map (EGM) on effectiveness of social protection measures in LMICs.

EGM provide a visual overview of the availability of evidence for a particular sector. In this EGM, we will include people in LMICs benefiting from social protection interventions. The EGM will consolidate what we know and do not know about “what works” by mapping out existing and ongoing systematic reviews and impact evaluations in this field; and by providing a graphical display of areas with strong, weak or nonexistent evidence on the effect of interventions or initiatives.

#### 1.1.2 Scope of the EGM

The EGM will include randomised controlled trials (RCTs), non‐RCTs and systematic reviews of effects of interventions. The map will be presented in two dimensions: the rows will list interventions and subcategories, and the columns will include the outcome domains. Each cell will include studies which contain evidence on the combination of intervention and outcome. Included studies will be coded for additional characteristics tas filters, such as population subgroup, context, country and region.

#### 1.1.3 Conceptual framework of the EGM

The intervention categories are based on the World Bank's Group 10‐year social protection and labour strategy, 2012–2022 (The World Bank, 2012) and the outcomes included in the map are based on these SDGs.

**Figure 1:**
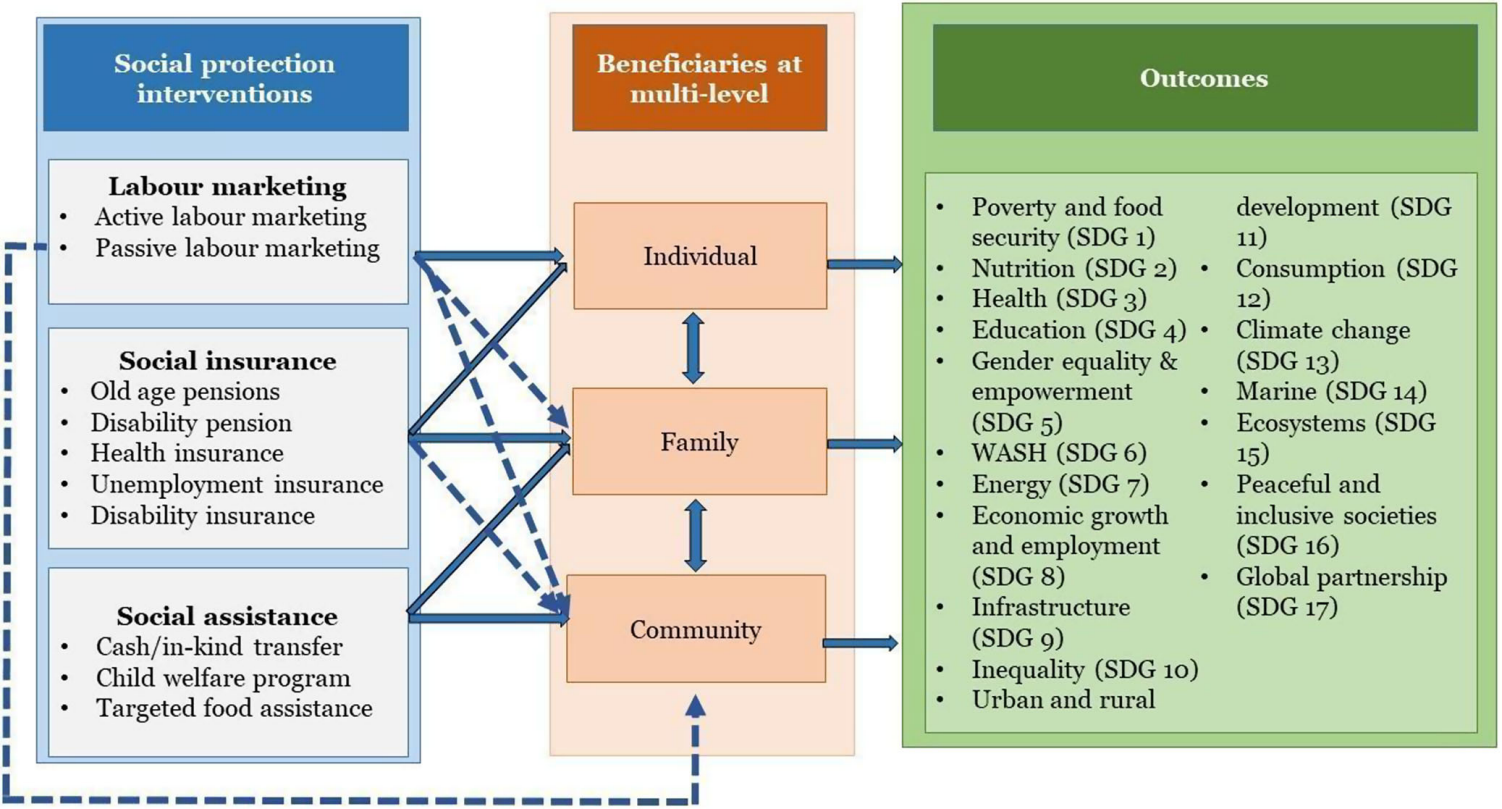
Conceptual framework on the effectiveness of social protection interventions in low‐ and middle‐income countries: an evidence and gap map

The World Bank (2012) emphasises that social protection and other interventions may buffer individuals from shocks and equip them to improve their livelihoods and create opportunities to build a better life for themselves and their families. Social protection and interventions like labour marketing, policies and programmes related to social insurance and social assistance are designed for individuals and families, and communities can be broadly transformed by providing a foundation for inclusive growth and social stability. Social protection and social assistance programmes listed in the intervention categories based on the World Bank's Group 10‐year social protection and labour strategy address chronic poverty. Their main goal is to protect poor individuals, families and communities at large from irreversible and catastrophic losses of human capital thereby contributing to equity through protecting against destitution and promoting equality of opportunity Figure 1 In addition, social protection and interventions provide broad‐based foundation for inclusive growth and social stability. Social protection interventions are centrally aimed at family and individual behaviour and thereby influence the behaviour of the whole community. Thus, social and cultural contexts affect their outcomes. Persistent shortfalls and the challenges have led to lack of progress by numerous countries in reaching the Millennium Development Goals (MDGs).

The MDG framework expired in 2015, since the UN General Assembly adopted “Transforming our World: The 2030 Agenda for Sustainable Development”, a resolution outlining a new framework to form the cornerstone of the sustainable development agenda for the period leading up to 2030 (UN, 2015). The SDGs have incorporated 17 universal goals and 169 targets. The SDGs substantially broaden the development agenda beyond the MDGs and are expected to frame UN member state policies over the next 15 years (IAEG‐SDGs, 2016).

The One‐UN Social Protection Floor initiative (SPF‐I), currently co‐led by the ILO and the World Health Organization (WHO) and endorsed by the UN Chief Executives Board, calls for an integrated set of social policies to provide income security and access to essential social services for all, paying particular attention to vulnerable groups. The core elements of the SPF‐I are: a basic set of social transfers, in cash and in kind, to provide a minimum income and/or employment and livelihood security for the unemployed and working poor the universal access to essential social services in the areas of health, water and sanitation, education, food security, housing and others defined by national priorities (ILO, 2005, 2011a, 2011b). As per Devereux and Sabates‐Wheeler (2004), in order to achieve SDGs, the first policy action SDG 1.3 is to “implement nationally appropriate social protection systems and measures for all and by 2030 to achieve substantial coverage of the poor and vulnerable”.

An important task is also to customise interventions to the beneficiaries at the individual, family and community levels. The timing and types of social protection interventions should be geared to meeting the specific needs and vulnerabilities especially with regard to protecting individuals, families and communities against health shocks or promoting livelihood ladders so that the SDGs are attained. If the SDGs that include social inclusion and gender equity, nutrition, employment, education, health and economic goals are to become a reality, social protection for all men and women and their families must be a key global objective. At the same time, for social protection to play an effective role in sustainable development its scope must be expanded to meet new global needs and to render it more effective and relevant to the world's entire population.

Social protection measures may have a potential to directly improve the well‐being of the individuals, families and thereby the community at large. Socioeconomic security is key to the well‐being of the individual and the families in the community. By responding to people's needs, social protection fosters social inclusion, gender equity and helps to build secure communities and stable societies thereby contributing to improvement in the SDGs.

Social protection has a strong focus on SDG outcomes like eradication of poverty and provision of food security. Social protections are important to end poverty and hunger as well as provide for food security in people both in developed and developing countries. In developing countries, the main emphasis of social protection is on addressing the causes of poverty, and not simply its symptoms, whereas in the developed countries the emphasis of social protection is on income maintenance and on protecting living standards for all (but especially workers). Social protection measures also ensure availability, sustainable management of water, sanitation, and they emphasise on ensuring that affordable, clean energy is available. Social protection measures also help in enhancing scientific research and upgradation of the technological capabilities of industrial sectors, thereby focusing on industry, innovation and infrastructure. Social protection interventions would also help in ensuring that safe, affordable housing and basic services are provided to people with disabilities, thus contributing in building sustainable cities and communities. Social protection measures also help in improving the sustainable pattern of consumption and production. Social protection measures help to some extent in protecting, restoring and promoting sustainable use of eco‐systems, manage forests, combat desertification, reverse land degradation and halt biodiversity loss. Additionally, social protection measures focus on mobilisation of financial resources for developing countries. Thus, the social protection interventions promote peaceful and inclusive societies for sustainable development at individual, family and community level. As stated in a social policy and development programme paper by United Nations Research Institute for Social Development (2010), as a policy framework, social protection interventions are key components for any development policies, addressing the SDGs and vulnerabilities in developing countries (Craig, 2011).

#### 1.1.4 Why it is important to develop the EGM

In response to above, there is an increased need for investment in generation of sound evidence on effective strategies for social protection to strengthen the policy architecture in this area. However, there are still many gaps in the evidence base from these programmes, including geographical gaps, thematic gaps and missing information that constrain the building of a concerted evidence‐informed policy and investment agenda. EGMs can contribute by providing an overview of available quality studies, identifying gaps and thereby aiding the prioritisation of global evidence synthesis needs and impact evaluations. The knowledge generated by the EGM will have the potential to support the identification of areas with potentially sufficient studies to conduct systematic reviews, and assist funders and policymakers to inform funding and/or policy decisions related to the social protection in LMICs. These can be decisions about the selection of potentially effective interventions or the funding of research—for example, research that can fill existing gaps in the knowledge base. This knowledge can facilitate the development of research agendas and priorities.

#### 1.1.5 Existing EGMs and/or relevant systematic reviews

There is no EGM specifically on social protection interventions in LMICs. However, an EGM titled, “Productive Safety Nets Gap Map” is available in 3ie (https://gapmaps.3ieimpact.org/evidence-maps/productive-safety-nets-gap-map-all-populations). The EGM provides the evidence on the effects of productive social safety net programmes on poverty and related outcomes. Our EGM focuses on social protection interventions using a broader approach.

There are a number of related systematic reviews available and some are listed below:

Systematic Reviews:

1. Interventions to improve the labour market for adults living with physical and/or sensory disabilities in LMICs: A systematic review September 2017.
2. Vocational and business training to improve women's labour market outcomes in LMICs: A systematic review. June 2018.
3. Interventions to improve the labour market outcomes of youth: A systematic review of training, entrepreneurship promotion, employment services and subsidised employment interventions 2017.
4. Disability and social protection programmes in low‐ and middle‐income countries: A systematic review.
5. Child protection training for professionals required to report child abuse and neglect. 2015.

## 2 OBJECTIVES

This EGM will provide an overview of the existing systematic reviews and impact evaluations on the key outcome domains and interventions aimed at improving social protection among people living in LMICs.

The specific objectives of this map are to:

1. Develop a clear framework of types of interventions and outcomes related to the effectiveness of interventions on social protection for people in LMICs
2. Map available systematic reviews and primary studies on the effectiveness of interventions on social protection for people in LMICs
3. Provide a structured and accessible collection of existing evidence and identifying gaps in the available evidence on social protection intervention, thereby helping to inform the research agendas of funders and other organisations.

## 3 METHODS

### 3.1 Defining EGMs

Mapping the evidence in an existing area is a relatively new approach that has been used since the early 2000s (Saran & White, 2018). EGMs are “evidence collections” (Snilstveit et al., 2013, p. 3) that provide a visual overview of the availability of evidence for a particular sector. They help to consolidate what we know and what we do not know about studies that evaluate the effectiveness of interventions in a given area—by mapping out existing and ongoing systematic reviews and impact evaluations in this field, and by providing a graphical display of areas with strong, weak or non‐existent evidence on the effect of such interventions.

Studies included in an EGM are identified through a comprehensive search of published and unpublished literature, which targets both completed and ongoing studies—the latter to help identify research in development, which might help fill existing evidence gaps.

The methods for conducting EGMs drawn on the principles and methodologies adopted in existing evidence mapping and synthesis products.

#### 3.1.1 Framework development and scope

The EGM framework will inform the inclusion and exclusion criteria of the EGM. Here, we describe the population, intervention, comparison, outcomes (indicators) and study designs for the map Table 1.

**Table 1:** Intervention categories, subcategories and examples

Intervention category	Subcategory	Examples
1. Social assistance	Cash transfer	Monthly transfer, individual transfer, family transfer
	In‐kind transfers	Food baskets, ration
	Child Welfare Programs	Mandatory education, nutritional supplement
	Targeted food assistance	Food stamps, food aid, school feeding,
2. Social insurance	Pensions (old age)	Defined contribution, defined benefit or carrier average
	Pensions (disability)	Disability work pension
	Health insurance	Individual insurance, family benefits, government insurance
	Unemployment insurance	Knowledge fair, skill education
	Disability insurance	disability monthly insurance, reservation
3. Labour marketing	Active labour market (job centres, training and policies to promote small and medium sized enterprises)	Technical skill training, business skill training, literacy/numeracy skill training, soft skill training, business advisory, access to markets and value chains, microfinancing, grants, job search assistance, public work programmes
	Passive labour market (maternity benefits, injury compensation, and sickness benefits, minimum wage etc.)	Autonomous health communities, unemployment insurance and unemployment assistance

##### Population

The key population of interest for this map is all the people living in LMICs.

LMICs are defined by World Bank as low‐income economies—those with a GNI <$995; lower middle‐income economies—those with a GNI per capita between $996 and $3895; and upper middle‐income economies—those with a GNI per capita between $3896 and $12,055 (2018). Both males and females in all the age group will be included in the EGM. The age group will be classified based on the WHO age criteria; infanthood (<3 years of age), childhood (3–11 years), adolescence (12–18 years), adults (19–59 years), elderly (61–80 years) and oldest (80+ years). Population subgroup of interest includes: vulnerable children (e.g., child labourers, orphans), men, women, ethnic minorities, conflict‐affected, people living with disabilities, the displaced, migrants, refuges and the unemployed.

##### Intervention

The intervention categories framework is based on the World Bank's Group 10‐year social protection and labour strategy (2012–2022). It identifies three evidence‐based strategies to improve social protection: social assistance, social insurance and labour marketing.

Table 1 lists the intervention categories and subcategories. Examples of programme names are given in brackets. These are listed to support the search and coding. The included interventions cover all main strategies to improve social protection. The subcategories of interventions and its definitions are given as Appendix A.

The intervention categories included in our map are:

1. Social assistance: noncontributory transfers in cash, vouchers, or in‐kind (including school feeding) to individuals or households in need, public works programmes; fee waivers (for basic health and education services); and subsidies (e.g., for food, fuel) (White, 2016)
2. Social insurance: contributory schemes providing compensatory support in the event of contingencies such as illness, injury, disability, death of a spouse or parent, maternity/paternity, unemployment, old age, and shocks affecting livestock/crops (White, 2016).
3. Labour marketing: active (promoting labour market participation) or passive (ensuring minimum employment standards) (White, 2016)
4. The social protection may include multiple interventions. For example, social assistance comprises of cash transfer, in‐kind transfer, children welfare activities and targeted food supply and this may be narrowed down further. For instance, food supply in the form of in‐kind transfer or cash transfer for food resources can improve people's nutritional status and it improves health of beneficiaries (Leroy et al., 2013). In‐kind transfer and cash transfer have positive effects on social financial, nutritional and health status of the public (Cunha et al., 2019). Governmental bodies, private contracts or charity trusts, may provide in‐kind transfer (Lieber & Lockwood, 2019). Similarly, children's well‐being is a multidimensional, complex and broad concept and this well‐being gives appropriate understanding of the child in diverse areas including health, material well‐being, education, conditions of housing and environment, and interpersonal relations (UNICEF, 2014). World Health Organization recommends financial support in addition to social health insurances to protect against financial risk and to improve access to healthcare services (WHO, 2005). Unemployment rates have increased since global financial crisis and in some countries it has deteriorated. As a result, unemployment protection policies, especially the unemployment insurance system, are a huge solution to address the problem (Stuckler & Basu, 2013)

##### Outcome

The seventeen main outcome categories are listed in the Table 2 given below

**Table 2:** Outcome domains

Poverty and food security (SDG 1)
Nutrition (SDG 2)
Health (SDG 3)
Education (SDG 4)
Gender equality and empowerment (SDG 5)
WASH (SDG 6)
Energy (SDG 7)
Economic growth and employment (SDG 8)
Infrastructure (SDG 9)
Inequality (SDG 10)
Urban and rural development (SDG 11)
Consumption (SDG 12)
Climate change (SDG 13)
Marine (SDG 14)
Ecosystems (SDG 15)
Peaceful and inclusive societies (SDG 16)
Global partnership (SDG 17)

SDG1: No Poverty: SDG1 aims at eradicating extreme poverty all over the world by 2030. Social protection measures are very important to end poverty and hunger among people with disabilities (https://www.un.org/development/desa/disabilities/envision2030-goal1.html).

Nutrition is one of the very essential needs of a human being. UNDP focuses on “zero hunger” (SDG 2) to have better access to safe, nutritious and sufficient food for all, reduce malnutrition, improve agricultural productivity and incomes of small‐scale food producers and opportunities for women and indigenous people to grow food and utilise their productions (https://www.un.org/sustainabledevelopment/hunger/). This directly contributes for social wellbeing.

Quality health and well‐being (SDG 3) is the base for our fifth outcome in this EGM. The aims of SDG 3 are reduce maternal mortality rate, preventable deaths of newborns and children, epidemics communicable (AIDS, tuberculosis, malaria, hepatitis, water‐borne diseases) and noncommunicable diseases, deaths and injuries from road traffic accidents and substance abuse. The goal also targets on improving access to health resources, utilisation of health services, health literacy, development of vaccines and medicines and health work force (https://www.un.org/sustainabledevelopment/health/).

The fourth outcome category in our EGM is education and it is based on “quality education” (SDG 4) of the UNDP SDGs. The intentions of SDG 4 are equitable and quality primary and secondary education leading to employment, access to quality early childhood development, access for all women and men to affordable and quality technical, vocational and tertiary education, including university, eliminate gender disparities in education, upgrade education facilities to child and disable and increase the scholarship and qualified teachers (https://www.un.org/sustainabledevelopment/education/).

Social inclusion and gender equity is based on “equity and gender” (SDG 5) and “reduced inequality” (SDG 10). The targets are reduction in child marriage, female discrimination, violence against women and child, harmful practices (genital mutilation, early/forced marriage). SDG 5 and SDG 10 also aim in improvement of education of girl child, leadership of women, access to sexual and reproductive health and reproductive rights, rights to economic resources/ownership to women, enabled use of technology, enforceable legislation for the promotion of gender equality, and recognise and value unpaid care and domestic work (https://www.un.org/sustainabledevelopment/gender-equality/).

SDG6: Clean water and sanitation: The main goal of SDG6 is to ensure availability and sustainable management of water and sanitation for all. The main targets are safe and affordable drinking water for all, sanitation and hygiene for all, improve the water quality, remove water scarcity and to save the water related eco‐systems (https://www.un.org/development/desa/disabilities/envision2030-goal6.html).

SDG7: Affordable and clean energy: SDG7 emphasise on ensuring modern energy services, and enhancing international collaboration in energy research and technology (https://www.un.org/development/desa/disabilities/envision2030-goal7.html).

Decent work and economic growth (SDG 8) is the bases for this outcome. It targets improving per capita economic growth, family income, achieve full and productive employment and decent work for all. In addition, growth of micro, small and medium‐sized enterprises, including access to financial services are included (https://www.un.org/sustainabledevelopment/economic-growth/).

SDG9: Industry, innovation and infrastructure: The goal of SDG9 is to build resilient infrastructure, promote inclusive and sustainable industrialisation and foster innovation. Enhancing scientific research and upgrading the technological capabilities of industrial sectors is also a target (https://www.un.org/development/desa/disabilities/envision2030-goal9.html).

Employment is one of the means to lead a satisfying life in any country. The 10th goal of UNPD is “reduced inequality” (SDG 10) concentrates on decent job creation, entrepreneurship, creativity and innovation, and encourage the formalisation and growth of micro‐, small‐ and medium‐sized enterprises. It targets full and productive employment and decent work for all women and men, including for young people and persons with disabilities, and equal pay for work of equal value, reduction of unemployment of youth. The other targets of SDG 10 are to eradicate forced labour, modern slavery and human trafficking and elimination of child labour and child soldiers and promote safe and secure working environments for all workers, including migrant workers, and expand access to banking, insurance and financial services for all (https://www.un.org/sustainabledevelopment/inequality/).

SDG 11: Sustainable cities and communities: The goal of SDG 11 is to make the cities and human settlements inclusive, safe, resilient and sustainable. One of the targets is to ensure safe and affordable housing and basic services and upgrade the slums. Improving public transport and road safety is also part of it (https://www.un.org/development/desa/disabilities/envision2030-goal11.html).

SDG12: Responsible consumption and production: SDG12 aims at sustainable management and efficient use of natural resources. Also emphasise on scientific disposal of chemicals and wastes. It also targets on sustainable pattern of consumption and production (https://www.un.org/development/desa/disabilities/envision2030-goal12.html).

SDG13: Climate action: SDG 13 focus on minimising the climate related hazards and natural disasters in different parts of the world. It also targets on integrating the climate change measures into national policies (https://www.un.org/development/desa/disabilities/envision2030-goal13.html).

SDG14: Life below water: SDG14 aims at reducing marine pollution. The main target is to protect the marine and coastal ecosystems. Emphasise on conserving marine, ocean and sea resources (https://www.un.org/development/desa/disabilities/envision2030-goal14.html).

SDG15: Life on land: SDG15 targets on protecting, restoring and promoting sustainable use of terrestrial ecosystems, sustainably manage forests, combat desertification, and halt and reverse land degradation and halt biodiversity loss (https://www.un.org/development/desa/disabilities/envision2030-goal15.html).

SDG16: peace and justice strong institutions: SDG16 targets on promoting peaceful and inclusive societies for sustainable development, provide access to justice for all and build effective, accountable and inclusive institutions at all levels. Focuses on reducing all types of violence and related death rate. It also aims at reducing corruption and bribery (https://www.un.org/development/desa/disabilities/envision2030-goal16.html).

SDG17: partnerships to achieve the goal: SDG17 aims at strengthening domestic resource mobilisation. Also focus on mobilising additional financial resources for developing countries. It also targets adopting and implementing investment promotion regimens (https://www.un.org/development/desa/disabilities/envision2030-goal17.html).

### 3.2 Stakeholder engagement

We will engage stakeholders on the evidence matrix at various organisations who provide Social protection sector policy and programmes. The framework will be informed by theory of change, relevant academic literature and consultations with key stakeholder, including research funders, implementing agencies, experts and researchers.

### 3.3 Data collection and analysis

#### 3.3.1 Screening and study selection

##### Types of study designs

Systematic reviews and impact evaluation or studies of effectiveness will be included for the EGM.

Definition of systematic reviews: According to the PRISMA definition for SRs (Moher et al., 2015) where the article explicitly states the methods used to identify studies (i.e. a search strategy), strategies for study selection (e.g., eligibility criteria and selection process) and explicitly detail methods of synthesis.

Impact evaluations are defined as intervention evaluations or field experiments that use quantitative approaches applied to experimental or observational data to measure the effect of an intervention relative to a counterfactual representing what would have happened to the same group in absence of that intervention (Khandker et al., 2009). Impact evaluations may also test different intervention designs. Specifically, we will include:

1. Studies where participants are randomly assigned to treatment and comparison group.
2. Studies where assignment to treatment and comparison group is based on other known allocation rules, including a threshold on a continuous variable (regression discontinuity designs). We will include: regression discontinuity designs, fixed effect estimation, instrumental variable, propensity score matching and difference‐in‐difference.
3. Since we anticipate a dearth of evidence in this sector from LMICs we will also include Before‐versus‐after studies with no comparison group.

*Treatment of qualitative research*

We do not plan to include qualitative research in the EGM.

#### 3.3.2 Types of settings

All types of settings in the LMICs (The World Bank, 2018) where interventions for social protection were implemented, will be included in the EGM. Examples of the types of settings will include village, town, district, state, urban, semiurban, rural, family, school, refugee camps, old age homes, country at large or a particular geographical or political locations, and so forth.

#### 3.3.3 Status of Studies

Relevant completed or ongoing studies will be included in the EGM.

The detailed eligibility criteria is given in Appendix C.

### 3.4 Search strategy and status of studies

The search for included studies will be conducted in three stages:

**Table:** 

Stage	Step	Timeline
Stage 1	Few of the included studies were pilot screened and coded	April 2019
Stage 2	We search of relevant systematic reviews and primary studies from appropriate databases and international organisations	March 2021
Stage 3	This stage will include search on additional websites for grey literature after expert consultation (in progress)	May 2021

The search will be as comprehensive as possible, using (but not limited to) relevant bibliographic databases, web‐based search engines, websites of specialist organisations, bibliographies of relevant reviews, and targeted calls for evidence using professional networks or public calls for submission of articles.

Additionally, citation searches of included studies in Google Scholar, Scopus and Web of science will be performed. Reference lists of the included reviews will be reviewed (Appendix B).

#### 3.4.1 Databases

1. Systematic review databases
  1. 3ie Systematic Review Database
  2. Campbell Collaboration
  3. Cochrane
  4. Collaboration for Environmental Evidence
  5. EPPI Centre Evaluation Database of Education Research
  6. PROSPERO
  7. Research for Development
  8. Swedish Agency FOR Health Technology Assessment and Assessment of Social Services
  9. Epistomonikos
2. Academic databases
  1. Applied Social Sciences Index and Abstracts (ASSIA)
  2. CABI's Global Health
  3. Caribbean Child Development Centre Online Publication Database—CINAHL
  4. EBSCO
  5. EBSCOhost (Caribbean Search)–Econlit–Eldis–Embase–Emerald insight–ERIC
  6. Google Scholar–International Bibliography of Social Sciences (IBSS)
  7. IDEAS‐Repec
  8. Popline–JGATE–JOLIS–JSTOR–MEDLINE–PsycINFO
  9. PubMed
  10. RedALyC (La Red de Revistas Científicas de América Latina, el Caribe, España y Portugal)
  11. SafetyLit
  12. SciELO–SCOPUS–Social Science Research Network (SSRN)–Sociological abstracts (ProQuest)–The National Bureau of Economic Research (NBER)–Web of Science–WHO's Global Health Library
3. International Organizations (Bilateral and multilateral)–DFID (including Research for Development (R4D)–ILO
  1. IOM–Save the Children–UN Women–UNDP–UNFPA Evaluation Database
  2. UNHCR–UNICEF
  3. UNICRI
  4. UNODOC–USAID–WHO/PAHO 4. Grey Literature search/websites–Abdul Latif Jameel Poverty Action Lab (J‐PAL)–Action against Hunger–Action Aid htt p–ADOLEC
  5. African Development Bank
  6. Africa‐Wide
  7. Africaportal.org-African journals online (AJOLS)–Anulib–Association for the Development of Africa–British Library for Development Studies
  8. CAF Development Bank of Latin America–CARE
  9. CEPAL/ECLAC—Economic Commission for Latin America and the Caribbean–Child and Youth Finance International–CIFF–Clinton Foundation–Concern Worldwide Division for Social Policy & Development
  10. Child Fund International
  11. Fórum Brasileiro de Segurança Pública
  12. CPC Learning Network
  13. EU CORDIS
  14. Gates Foundation–GreyNet International–Innovations for Poverty Action (IPA) Database
  15. International Center for Research on Women (ICRW)
  16. Inter‐American Development Bank (IADB)
  17. International Food Policy Research Institute (IFPRI)–International Rescue Committee (IRC)–International Red Cross–IPC‐IG (Working papers)–Joanna Briggs Institute Evidence‐Based Practice Database
  18. LLC/Centre for Human Services
  19. LTSHM–MedCarib
  20. Medecins Sans Frontières–Oak Foundation
  21. One International–Opengrey
  22. Organization of American States (OAS)
  23. Overseas Development Institute–Project Concern–Proquest Dissertations & Theses
  24. RAND Corporation
  25. Sexual Violence Research Initiative (SVRI)–Social Care Online–UN Economic and Social Council UNESCO–UNICEF Innocenti Research Centre
  26. University Research Co.–United Nations Population Fund–Urban Youth Evidence Synthesis–Valid International–Working Group on Early Childhood Development–World Bank Group (WBG)
  27. Within WBG: Spanish Impact Evaluation Fund (SIEF)
  28. Within WBG: Korean Trust Fund (KTF)–World Food Programme–World for World Organization
  29. World Vision

### 3.5 Screening and selection of studies

All titles and abstracts, and then full text, will be double screened. A third reviewer will help to resolve in the event of disagreement. The screening tool is given as Appendix D.

### 3.6 Data extraction, coding and management

Coding will be done independently by two coders, with a third‐party arbitrator or in the event of disagreement.

### 3.7 Data extraction and management

State how data will be extracted from reports of included studies, clarifying how many people will be involved (and whether independently), whether machine learning will be used (and if so, how) and how disagreements will be handled (ER34). Campbell reviews usually use two independent reviewers to screen for inclusion. However, there is increasing use of automation. Any use of automation and text‐mining should be described in sufficient detail for replication and with information about validity and testing of the method.

List the types of information that will be sought from reports of included studies (ER36). Describe any attempts to obtain or clarify data from individuals or organisations, if applicable (ER35).

Describe what coding categories will be used, how they will be collected and by whom. Coding sheets should be pretested and included as an annex in the protocol.

### 3.8 Quality appraisal

The quality of the included systematic reviews will be assessed using AMSTAR 2 and done independently by two reviewers. The quality review of the primary studies will be rated based on the quality assessment tool for individual studies as described below.

The tool used to assess study quality is shown in Appendix E. This tool includes six criteria that are appropriate for the assessment of quantitative impact evaluations. These are as follows:

1. *Study design (potential confounders taken into account)*: impact evaluations need either a well‐designed control group (preferably based on random assignment) or an estimation technique which controls for confounding and the associated possibility of selection bias.
2. *Power calculations*: Small sample size can result in an under‐powered study with a high risk of not detecting an effect from the intervention when there actually is one. The combination of under‐powered studies and publication bias can put an upward bias on the assessment of the overall effect size from a body of literature. The problem of sample size is addressed by conducting power calculations before the study to determine the required sample size. We will not use this item in the overall assessment of the study. However coding mention of power calculations signals the importance of both conducting and reporting power calculations.
3. *Attrition or losses to follow up*: can be a major source of bias in studies, especially if there is differential attrition between the treatment and comparison group (called the control group in the case of RCTs) so that the two may no longer be balanced in preintervention characteristics. The US Institute of Education Sciences What Works Clearing House (WWC) has developed standards for acceptable levels of attrition, in aggregate and the differential, which are applied here.
4. *Description of intervention*: If the intervention is not well described then the evidence may be misinterpreted to support an intervention not actually supported by study findings. For example, “case work” or “shelter” are very broad descriptions, so more detail of the intervention is required so as to know what is actually being evaluated. We rate as low if the description is just named with no description, medium if there is a short description, and high if there is a detailed description. We do not use this item in the overall assessment of the study.
5. *Definition of outcomes*: Outcomes should be clearly defined so that study findings can be properly interpreted. So far as possible, unless a subjective perception is required, that questions should rely on objective factors, and utilise data collection instruments which have been validated for the context in which they are being applied. We rate as high if there is a clear definition of the outcome and how it is being measured, or reference to an existing tool. Medium rating is given is if there is a brief description, and low if the outcome is named but not adequately described.
6. *Baseline balance*: Baseline balance means that the treatment and comparison groups have the same average characteristics at baseline, not only for outcomes but other factors which may affect the impact of the programme.

*Overall assessment*: The overall assessment uses a weakest link in the chain principle so that the overall assessment is the lowest of assessment given to any of the relevant items. As mentioned above, not all items are used in this assessment. So the overall assessment is the lowest of the assessments for items 1, 4, 6 and 7.

### 3.9 Planned analysis

The EGM report shall provide tabulations or graphs of the number of studies, with accompanying narrative description, by

- Intervention category and subcategory
- Outcome domain and subdomain
- Table of aggregate map of interventions and outcomes
- Region
- Year
- Study type
- Population subgroups

#### 3.9.1 Unit of analyses

Each entry in the map will be a systematic review or a primary study of effectiveness. The final EGM will identify the number of studies covered by the map in each sector or subsector.

#### 3.9.2 Presentation

In addition to the interventions and outcomes, the following filters will be coded:

1. Population subgroups: The age group is classified based on the WHO age criteria stated as follows: infanthood (<3 years of age). childhood (3–11 years), adolescence (11–18 years), adults (18–60 years), elderly (above 60 years), boys/men, girls/women, pregnant women/girls, children with disability, children in low income settings, ethnic minorities.
2. Context: Very high prevalence setting such as poor and vulnerable, children, women, older people, and people living with disabilities, the displaced, the unemployed, the sick, male, female, LGBTQ, father, mothers, elderly, migrants, adolescents, and adults.
3. Region: East Asia and Pacific, Europe and Central Asia, Latin America and Caribbean, Middle East and North Africa, North America, South Asia, Western Central Africa, Eastern Central Africa.
4. Conflict‐affected regions: This will be defined based on Fragile and Conflict Affected State (FCAS) list of conflict affected regions updated as per current year (2018/2019) as high, medium, low, neighbours and non‐conflict affected region (UNDP).

## CONTRIBUTIONS OF AUTHORS

Please note that this is the *recommended optimal* EGM team composition.

- Content
  Dr Latha holds PhD in Medical surgical nursing and has been extensively involved in areas of oncology, nursing Education and Research. She also published various research articles on nursing and medical research.
- EGM methods
  Ashrita Saran has previous experience in systematic review methodology, including searching, data collection, and theory‐based synthesis, which means she is proficient in carrying out the various processes in an EGM, such as search, eligibility screening, quality assessment and coding. She has undertaken an overview of approaches to mapping in a range of organisations. All the authors have previous experience of working on various stages of systematic reviews as screening, coding, drafting search strategy.
- Information retrieval
  Ashrita Saran and Latha are trained in designing and implementing search strategies.

All the authors have previous experience of drafting search strategy and have performed systematic review searches.

## Declarations of interest

No conflict of interest.

### Plans for updating the EGM

Once completed, the EGM will be updated every three years. The lead author and/or the corresponding authors will be responsible for updating the EGM.

## Differences between protocol and review

Explain and justify any changes from the protocol (including any *post hoc* decisions about eligibility criteria (ER59).

## Figures and Tables

**Figure 2:**
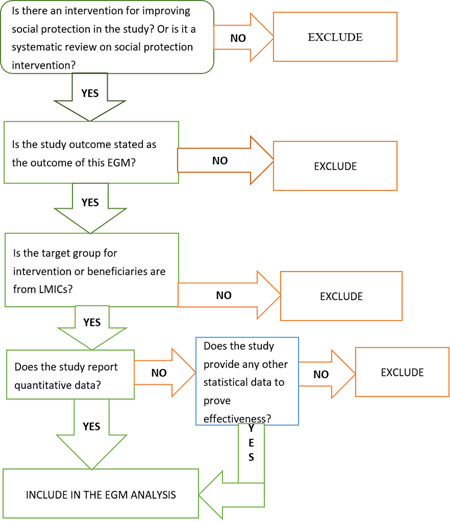
Screening tool for the effectiveness of social protection interventions in low‐ and middle‐income countries: an evidence and gap map

## APPENDIX A DEFINITION

1. Social Security: Social security is a set of measures and policies that help all households and individuals either avoid or cope with financial difficulties during their lifetime. Social security is foremost a human right embedded in the Universal Declaration of Human Rights, 1948 (Article 22) (General Assembly of the United Nations, 1948)

Social security is the protection that a society provides for its members: (i) to compensate for the loss of income caused by one of these contingencies (for instance, financial support when you are unemployed) and (ii) to facilitate access to social services (such as health services and education) and fulfil basic needs (International Labour Organization, 2017).

1. Social/cash transfer/In‐Kind transfers: Cash transfers: are direct, regular and predictable transfers that raise and smooth incomes to reduce poverty and vulnerability (Arnold et al., 2011, p. 2). Unconditional Cash Transfers (UCTs) are for the beneficiary to decide how to spend. Conditional Cash Transfers (CCTs) are given with the requirement that the beneficiary meets certain conditions—often related to human capital development, such as visiting a health clinic or ensuring children go to school.
2. In‐kind transfers: are economic and livelihood asset transfers to households, facilitating income generation. They tend to be larger, one‐off transfers but can also be smaller, regular transfers, such as food transfers. They tend to take an integrated approach, linking the transfer with skills training and other activities (Holmes & Jones, 2013, p. 65) (https://gsdrc.org/topic-guides/social-protection/types-of-social-protection/)
3. Child Welfare Programs: Child welfare is a continuum of services designed to ensure that children are safe and that families have the necessary support to care for their children successfully. Child welfare agencies typically:

- Support or coordinate services to prevent child abuse and neglect
- Provide services to families that need help protecting and caring for their children
- Receive and investigate reports of possible child abuse and neglect; assess child and family needs, strengths, and resources
- Arrange for children to live with kin (i.e., relatives) or with foster families when safety cannot be ensured at home
- Support the well‐being of children living with relatives or foster families, including ensuring that their educational needs are addressed
- Work with the children, youth, and families to achieve family reunification, adoption, or other permanent family connections for children and youth leaving foster care
  (https://www.childwelfare.gov/pubPDFs/cw_educators.pdf)

1. Targeted Food Assistance: The Food and Nutrition Service (FNS) works to end hunger and obesity through the administration of 15 federal nutrition assistance programmes including WIC, Supplemental Nutrition Assistance Program, and school meals (https://www.nutrition.gov/food-assistance-programs)

1. Social insurance: A public insurance programme that provides protection against various economic risks (e.g., loss of income due to sickness, old age, or unemployment) and in which participation is compulsory. Social insurance is considered to be a type of social security (*q.v*.), and in fact the two terms are sometimes used interchangeably (https://www.britannica.com/topic/social-insurance)
2. Pension: A private or government fund (or payments therefrom), from which intermittent and regular benefits or allowances are paid to a person upon his or her retirement or disability (Duhaime's Law Dictionary)

Definitions of Pension (Justice Dumoulin, Exchequer Court of Canada)

- “A pension is an annuity or other periodical payment made, especially by a government, a company, or an employer of labour, in consideration of past services or of the relinquishment of rights, claims, or emoluments”.
- “Pensions are universally construed as a reward for long‐continued service paid upon retirement from service, and all pensions of public employees are paid upon their retirement”.
- “A pension is a stated allowance or stipend made in consideration of past services or of surrender of rights or emoluments to one retired from service” http://www.duhaime.org/LegalDictionary/P/Pension.aspx
- Types of Pension (State Pension Department, UK) https://www.pensionwise.gov.uk/en/pension-types
  a. Defined contribution/money purchase pensions—based on how much money has been paid into pension that are put into investments by the pension provider.
  b. Defined benefit (final salary or career average)—based on one's salary and how long one is working for one's employer.

1. Health Insurance: Insurance against loss through illness of the insured especially insurance providing compensation for medical expenses (Merriam Webster's Dictionary).

It is an arrangement in which one makes regular payments to an insurance company in exchange for that company paying most or all of the costs of one's medical care (Cambridge Dictionary).

It is a type of insurance that essentially covers your medical expenses (Insurance Regulatory and Development Authority [IRDA]) https://www.nhp.gov.in/sites/default/files/pdf/health_insurance_handbook.pdf

1. Labour market policy: Labour market policies (LMP) comprise all kinds of regulative policies that influence the interaction between labour supply and demand. They consist of polices that provide income replacement (usually called passive labour market policies) as well as labour market integration measures available to unemployed or those threatened by unemployment (International Labour Organization) https://www.ilo.org/empelm/areas/labour-market-policies-and-institutions/lang--en/index.htm
2. Passive labour market policy: In order to explain employment, the amount of unemployment benefits per unemployed (passive labour market policy) and payment for wage subsidies and training per employed and unemployed person (active labour market policy) are used in addition to real wages and output. Wages and output have their expected impact on total employment. It turns out that passive labour market policy has a negative, and active labour market measures a positive, effect on the number of persons employed. (https://www.tandfonline.com/doi/abs/10.1080/000368498325480).

Usually the unemployed, but also the underemployed and the employed who are looking for better jobs. So‐called “passive” policies are concerned with providing replacement income during periods of joblessness or job search (courses.itcilo.org/A358127/library/19-10-2015-09-38-49/at%26/AttachmentFile *)*

1. Active Labour Market Policy: Active labour market policies (ALMP) are tools addressed to either increase the probability of reemployment of unemployed workers or to reduce the probability of losing the job in the case of employed workers (Cueto & Patricia, 2014; “A review of active and passive labour market policies in Spain”, MPRA Paper 60648, University Library of Munich, Germany) https://mpra.ub.uni-muenchen.de/60648/1/MPRA_paper_60648.pdf
2. Unemployment protection is a means of protecting workers and their families against the loss of employment or earnings. The objectives of ILO for unemployment protection were to provide income security to protect unemployed workers and their families against poverty (through unemployment benefits); and increase employability through skills training and retraining, and facilitate the return to employment as soon as possible (through active labour market policies).

The ILO approach to unemployment protection covers: (International Labour Organization, 2017). Unemployment protection: A good practices guide and training package. ILO Regional Office for Asia and the Pacific).

comprehensive social protection, to provide income security or income replacement;

- Periodical and predictable benefits
- Facilitation of active search for work by linking it with other public policies, including employment
- Policies
- Promotion of employment, including Active Labour Market Policy (ALMPs) to support jobseekers and employers and
- Close coordination between unemployment protection and employment promotion policies.

1. Disability insurance: It relates to provision of disability‐specific programmes or initiatives aimed at overcoming particular disadvantages or barriers, as well as to ensure the inclusion of disabled persons in mainstream services and activities, such as skills training, employment promotion, social protection schemes and poverty reduction strategies. ILO Disability Inclusion Strategy and Action Plan 2014–2017; https://www.ilo.org/global/topics/disability-and-work/WCMS_475650/lang--en/index.htm
2. Maternity benefits refer to special protection to expectant and nursing mothers to prevent harm to their or their infants' health, adequate time to give birth, to recover, and to nurse their children. At the same time, protection to ensure that they will not lose their job simply because of pregnancy or maternity leave, so that it ensures a woman's equal access to employment, continuation of often‐vital income, which is necessary for the well‐being of expectant/nursing mothers’ entire family. International Labour Standards on Maternity protection. Maternity Protection Convention, 2000 (No. 183)—[ratifications] https://www.ilo.org/global/standards/subjects-covered-by-international-labour-standards/maternity-protection/lang--en/index.htm
3. Provident fund is a tailor‐made pension mechanism allowing a wide range of unorganised sector workers to access old‐age benefits. It aims at an extensive coverage encompassing both workers employed in small industries as well as the self‐employed engaged in various occupations. In order to encourage enrolment, the scheme provides for a matching contribution from the State. https://www.social-protection.org/gimi/ShowRessource.action?id=6581; https://www.ilo.org/wcmsp5/groups/public/---ed_protect/--soc_sec/documents/publication/wcms_secsoc_6581.pdf
4. Social inclusion (SDG 5): The World Bank Group defines social inclusion as the process of improving the terms for individuals and groups to take part in society, and the process of improving the ability, opportunity, and dignity of those disadvantaged on the basis of their identity to take part in society (https://www.worldbank.org/en/topic/social-inclusion )
5. Gender equity (SDG 10): Gender equity refers to the different needs, preferences and interests of women and men. This is often referred to as substantive equality (or equality of results) and requires considering the realities of women's and men's lives that involve strategies needed to reduce gender‐based social inequities (https://www.who.int/gender-equity-rights/knowledge/glossary/en/)
6. Nutrition (SGD 2): Nutrition is the intake of food, considered in relation to the body's dietary needs and necessary for health and growth. Poor nutrition can lead to reduced immunity, increased susceptibility to disease, impaired physical and mental development, and reduced productivity.
7. Employment (SGD 8): A contract in which one person, the employee agrees to perform work for another, employer. The employees comprises of two main groups; persons employed, at work and persons employed, not at work due to temporary absence from the job (https://www.insee.fr/en/metadonnees/definition/c1159)
8. Education: education is the process of educating or of being educated and resulting in improvement of knowledge.
9. Health and nutrition (SGD 3): World Health Organisation defines health as a “state of complete physical, mental, and social well‐being, and not merely the absence of disease or infirmity.” Health is a dynamic condition resulting from a body's constant adjustment and adaptation in response to stresses and changes in the environment for maintaining an inner equilibrium called homoeostasis (http://www.businessdictionary.com/definition/health.html). In the current EGM, we will include all the outcomes of social protection interventions related to promotional or preventive activities related to health
10. Economic (SDG 8): UN Definition ‐ Sustain per capita economic growth in accordance with national circumstances and, in particular, at least 7 per cent gross domestic product growth per annum in the least developed countries (https://sdg-tracker.org/economic-growth).

## APPENDIX B SEARCH STRATEGIES

Database: Ovid MEDLINE(R) ALL <1946 to June 20, 2019>

Search Strategy:

‐‐‐‐‐‐‐‐‐‐‐‐‐‐‐‐‐‐‐‐‐‐‐‐‐‐‐‐‐‐‐‐‐‐‐‐‐‐‐‐‐‐‐‐‐‐‐‐‐‐‐‐‐‐‐‐‐‐‐‐‐‐‐‐‐‐‐‐‐‐‐‐‐‐‐‐‐‐‐‐

1 social assistance.mp.

2 Social Support/

3 cash transfer.mp.

4 money transfer.mp.

5 currency transfer.mp.

6 Money lending.mp.

7 Cash lending.mp.

9 Financial incentives.mp.

10 Child Welfare Programs.mp.

11 Child* well‐being.mp.

12 Child* benefit.mp.

13 Social benefit for child.mp.

14 Targeted food assistance.mp.

20 food assistance.mp.

22 Food Assistance/

24 mid day meal.mp.

25 mid‐day meal.mp.

26 school* lunch*.mp.

27 food supply.mp. or Food Supply/

28 social insurance.mp.

29 social security.mp. or Social Security/

30 social allowance.mp.

31 social coverage.mp.

32 social assurance.mp.

39 Pensions/

40 old age pension.mp.

41 elderly pension.mp.

42 disability pension.mp.

43 financial support.mp. or Financial Support/

44 monetary support.mp.

45 superannuation.mp.

46 financial empower*.mp.

47 passive employ* program*.mp.

48 passive labour market.mp.

49 Unemployment insurance.mp.

50 unemployment support.mp.

51 matern* benefit.mp.

54 active labour market.mp.

55 job center.mp.

56 job centre.mp.

57 job centre*.mp.

58 job center*.mp.

63 sick* benefit*.mp.

67 minimum wage*.mp.

68 minimum salar*.mp.

69 minimum pay*.mp.

70 comparative study.mp. or Comparative Study/

71 Controlled Before‐After Studies/

72 Interrupted Time Series Analysis/

73 Prospective Studies/

74 Cohort Studies/

75 Meta‐Analysis/

78 meta analysis.mp.

79 placebo.mp.

81 (developing countr* or developing nation* or developing population* or "developing world" or less developed countr* or less developed nation* or less developed population* or "less developed world" or lesser developed countr* or lesser developed nation* or lesser developed population* or "lesser developed world" or under developed countr* or under developed nation* or under developed population* or "under developed world" or underdeveloped nation* or underdeveloped population* or "underdeveloped world" or middle income countr* or middle income nation* or middle income population* or low income countr* or low income nation* or low income population* or lower income countr* or lower income nation* or lower income population* or underserved countr* or underserved nation* or underserved population* or "underserved world" or under served countr* or under served nation* or under served population* or "under served world" or deprived countr* or deprived nation* or deprived population* or "deprived world" or poor countr* or poor nation* or poorer countr* or poorer nation* or poorer population* or developing economy* or less developed econom* or lesser developed econom* or under developed econom* or underdeveloped econom* or middle income econom* or low income econom* or lower income econom* or "low gdp" or "low gnp" or "low gross domestic" or "lower gdp" or "lower gnp" or "lower gross domestic" or "lower gross national" or lmic or lmics or "third world" or lami countr* or transitional countr* or L&MIC or LAMIC or LDC).mp. [mp=title, abstract, original title, name of substance word, subject heading word, floating subheading word, keyword heading word, organism supplementary concept word, protocol supplementary concept word, rare disease supplementary concept word, unique identifier, synonyms]

82 (Africa or Algeria or Angola or Benin or Botswana or Burkina Faso or Burundi or Cameroon or "Cape Verde" or "Central African Republic" or Chad or "Democratic Republic of the Congo" or "Republic of the Congo" or Congo or "Cote d'Ivoire" or "Ivory Coast" or Djibouti or Egypt or "Equatorial Guinea" or Eritrea or Ethiopia or Gabon or Gambia or Ghana or Guinea or Guinea‐Bissau or Kenya or Lesotho or Liberia or Libya or Madagascar or Malawi or Mali or Mauritania or Morocco or Mozambique or Namibia or Niger or Nigeria or Rwanda or "Sao Tome" or Principe or Senegal or "Sierra Leone" or Somalia or Somaliland or "South Africa" or "South Sudan" or Sudan or Swaziland or Tanzania or Togo or Tunisia or Uganda or Zambia or Zimbabwe).mp. [mp=title, abstract, original title, name of substance word, subject heading word, floating subheading word, keyword heading word, organism supplementary concept word, protocol supplementary concept word, rare disease supplementary concept word, unique identifier, synonyms]

83 ("South America" or "Latin America" or "Central America" or Mexico or Argentina or Bolivia or Brazil or Chile or Colombia or Ecuador or Guyana or Paraguay or Peru or Suriname or Uruguay or Venezuela or Belize or "Costa Rica" or "El Salvador" or Guatemala or Honduras or Nicaragua or Panama).mp. [mp=title, abstract, original title, name of substance word, subject heading word, floating subheading word, keyword heading word, organism supplementary concept word, protocol supplementary concept word, rare disease supplementary concept word, unique identifier, synonyms]

84 ("Middle East" or "South‐East Asia" or "Indian Ocean Island*" or "South Asia" or "Central Asia" or Caucasus or Afghanistan or Azerbaijan or Bangladesh or Bhutan or Burma or Cambodia or China or Georgia or India or Iran or Iraq or Jordan or Kazakhstan or Korea or "Kyrgyz Republic" or Kyrgyzstan or Lao or Laos or Lebanon or Macao or Mongolia or Myanmar or Nepal or Oman or Pakistan or Russia or "Russian Federation" or "Saudi Arabia" or Bahrain or Indonesia or Malaysia or Philippines or Sri Lanka or Syria or "Syrian Arab Republic" or Tajikistan or Thailand or Timor‐Leste or Timor or Turkey or Turkmenistan or Uzbekistan or Vietnam or "West Bank" or Gaza or Yemen or Comoros or Maldives or Mauritius or Seychelles).mp. [mp=title, abstract, original title, name of substance word, subject heading word, floating subheading word, keyword heading word, organism supplementary concept word, protocol supplementary concept word, rare disease supplementary concept word, unique identifier, synonyms]

85 ("Pacific Islands" or "American Samoa" or Fiji or Guam or Kiribati or "Marshall Islands" or Micronesia or New Caledonia or "Northern Mariana Islands" or Palau or "Papua New Guinea" or Samoa or "Solomon Islands" or Tonga or Tuvalu or Vanuatu).mp. [mp=title, abstract, original title, name of substance word, subject heading word, floating subheading word, keyword heading word, organism supplementary concept word, protocol supplementary concept word, rare disease supplementary concept word, unique identifier, synonyms]

86 ("Eastern Europe" or Balkans or Albania or Armenia or Belarus or Bosnia or Herzegovina or Bulgaria or Croatia or Cyprus or "Czech Republic" or Estonia or Kosovo or Latvia or Lithuania or Macedonia or Malta or Moldova or Montenegro or Romania or Serbia or "Slovak Republic" or Slovakia or Slovenia or Ukraine).mp. [mp=title, abstract, original title, name of substance word, subject heading word, floating subheading word, keyword heading word, organism supplementary concept word, protocol supplementary concept word, rare disease supplementary concept word, unique identifier, synonyms]

87 (((Afghanistan or Albania or Algeria or American Samoa or Angola or Armenia or Azerbaijan or Bangladesh or Belarus or Belize or Benin or Bhutan or Bolivia or Bosnia) and Herzegovina) or Botswana or Brazil or Bulgaria or Burkina Faso or Burundi or Cabo Verde or Cambodia or Cameroon).mp. [mp=title, abstract, original title, name of substance word, subject heading word, floating subheading word, keyword heading word, organism supplementary concept word, protocol supplementary concept word, rare disease supplementary concept word, unique identifier, synonyms]

88 (Central African Republic or Chad or China or Colombia or Comoros or Congo Dem* Rep* or Congo Rep* or Costa Rica or Cote dIvoire or Cuba or Djibouti or Dominica or Dominican Republic or Ecuador or Egypt Arab Rep* or El Salvador or Equatorial Guinea or Eritrea or Ethiopia or Fiji or Gabon or Gambia The or Georgia or Ghana or Grenada or Guatemala or Guinea or Guinea‐Bissau or Guyana or Haiti or Honduras or India).mp. [mp=title, abstract, original title, name of substance word, subject heading word, floating subheading word, keyword heading word, organism supplementary concept word, protocol supplementary concept word, rare disease supplementary concept word, unique identifier, synonyms]

89 (Indonesia or Iran Islamic Rep* or Iraq or Jamaica or Jordan or Kazakhstan or Kenya or Kiribati or Korea Dem* Peoples Rep* or Kosovo or Kyrgyz Republic or Lao PDR or Lebanon or Lesotho or Liberia or Libya or Macedonia FYR or Madagascar or Malawi or Malaysia or Maldives or Mali or Marshall Islands or Mauritania or Mauritius or Mexico).mp. [mp=title, abstract, original title, name of substance word, subject heading word, floating subheading word, keyword heading word, organism supplementary concept word, protocol supplementary concept word, rare disease supplementary concept word, unique identifier, synonyms]

90 (((Micronesia Fed* Sts* or Moldova or Mongolia or Montenegro or Morocco or Mozambique or Myanmar or Namibia or Nauru or Nepal or Nicaragua or Niger or Nigeria or Pakistan or Papua New Guinea or Paraguay or Peru or Philippines or Romania or Russian Federation or Rwanda or Samoa or Sao Tome) and Principe) or Senegal or Serbia or Sierra Leone or Solomon Islands or Somalia or South Africa or South Sudan or Sri Lanka).mp. [mp=title, abstract, original title, name of substance word, subject heading word, floating subheading word, keyword heading word, organism supplementary concept word, protocol supplementary concept word, rare disease supplementary concept word, unique identifier, synonyms]

91 (((((St Lucia or St Vincent) and the Grenadines) or Sudan or Suriname or Swaziland or Syrian Arab Republic or Tajikistan or Tanzania or Thailand or Timor‐Leste or Togo or Tonga or Tunisia or Turkey or Turkmenistan or Tuvalu or Uganda or Ukraine or Uzbekistan or Vanuatu or Venezuela RB or Vietnam or West Bank) and Gaza) or Yemen Rep* or Zambia or Zimbabwe).mp. [mp=title, abstract, original title, name of substance word, subject heading word, floating subheading word, keyword heading word, organism supplementary concept word, protocol supplementary concept word, rare disease supplementary concept word, unique identifier, synonyms]

92 1 or 2 or 3 or 4 or 5 or 6 or 7 or 9 or 10 or 11 or 12 or 13 or 14 or 20 or 22 or 24 or 25 or 26 or 27 or 28 or 29 or 30 or 31 or 32 or 39 or 40 or 41 or 42 or 43 or 44 or 45 or 46 or 47 or 48 or 49 or 50 or 51 or 54 or 55 or 56 or 57 or 58 or 63 or 67 or 68 or 69 (115240)

93 70 or 72 or 73 or 74 or 75 or 78 (2579504)

94 81 or 82 or 83 or 84 or 85 or 86 or 87 or 88 or 89 or 90 or 91 (1674375)

95 92 and 93 and 94 (1643)

***************************

## APPENDIX C DETAILED ELIGIBILITY CRITERIA

**Table:**

	Include	Exclude
Literature type	Published journal articles Grey literature including technical reports which report quantitative data related to effectiveness of interventions for violence against children Technical reports/working papers	Commentary or conceptual papers Editorial Conference proceedings Case studies
Study design	All study designs must include quantitative data Permissible study designs include: Systematic reviews Metanalysis/meta regressions Randomised controlled trial Modelling with empirically grounded parameters/Econometric studies (Regression discontinuity, Propensity score or other matching techniques, Difference in difference) Instrumental variables Other matching designs Rigorous quasi‐experimental design/Quasi‐experimental Natural experiments Single‐subject design Analytical observational Before‐after studies Time‐series	Purely qualitative studies will be excluded regardless of design. Descriptive studies (cross‐sectional studies) reporting data narratively, but do not give statistical analysis Literature reviews
Population	We will include all the people living in LIMCs including Infanthood, Childhood, Adolescence, Adults, elderly, boys/men, girls/women, pregnant women/girls, children with disability, children in low income settings, ethnic minorities, vulnerable children (e.g., child laborers, orphans), ethnic minorities, conflict‐affected, people living with disabilities, the displaced, migrants, refuges and the unemployed We will include multi‐centric studies provided the interventions are directed towards people of LMICs and a clear methodology is available. For systematic reviews with global focus, we will: Exclude the Systematic reviews that include studies only from High income even if they did not have any search restrictions	Mixed population from high, low‐middle and low income countries
Interventions	We will include studies with interventions that aim in improving social protection as a primary focus.	Interventions that focus on psychological wellbeing and human rights will be excluded
	1. Social assistance (Social safety nets): Social/Cash transfer/In‐kind transfers, Child Welfare Programs, Targeted food assistance 2. Social insurance: Pensions (old age/disability), Health Insurance. Passive labour market programs, Unemployment insurance, Disability insurance, Maternity benefits, Provident funds 3. Labour marketing: Active labour market, Job centres, Job training, Policies to promote small and medium sized enterprises, Passive labour market, Maternity benefits, Injury compensation, Sickness benefits, Minimum wage	
Outcomes	We will include studies that aim to social protection among people living in LMICs and including following:	1.
	2. Social inclusion and gender equity 3. Nutrition 4. Employment 5. Education 6. Health and nutrition 7. Economic	

## APPENDIX D SCREENING TOOL

Figure 2

## APPENDIX E CRITICAL APPRAISAL TOOL

**Table:**

Item		Point in time (where applicable)	Rating
1a	Study design (Potential confounders taken into account)	End of intervention	High confidence: RCT, RDD, ITT, instrumental variable Medium confidence: DiD with matching, PSM Low confidence: other matching
1b	Study design (Potential confounders taken into account)	Longest follow up (if applicable)	Study design may change at post end line follow up, usually loss of RCT as control becomes treated. Same codes as 1a
2	Masking or blinding (RCTs only)		High confidence: any blinding or any mention of blinding Medium confidence: no blinding Low confidence is not used for this item
3	Power calculations are reported		High confidence: any mention of power calculations as basis for sample size Medium confidence: no mention of power calculations Low confidence is not used for this item
4a	Losses to follow up are presented and acceptable*	End of intervention	High: attrition within IES bounds Medium: attrition close to IES bounds Low: attrition not reported or attrition outside IES bounds N/A for ex post studies
4b	Losses to follow up are presented and acceptable*	Longest follow up (if applicable)	High: attrition within IES bounds Medium: attrition close to IES bounds Low: attrition not reported or attrition outside IES bounds N/A for ex post studies
5	Intervention if clearly defined		High confidence: intervention clearly and fully described Medium confidence: brief description of interventionLow confidence: intervention named but not described, or not named
6	Outcome measures are clearly defined and reliable		High confidence: outcome measure clearly and fully described, preferably with reference to validation Medium confidence: brief description of outcome Low confidence: outcome named but not described
7	Baseline balance (N.A. for before versus after)		High confidence: RCT or baseline balance report and satisfactory (imbalance on 5 or <5%) Medium confidence: Imbalance between 5‐10 percent Low confidence: Baseline balance not reported, or reported and lack of balance on 10 or more than 10 percent
	Overall confidence in study findings	End of intervention	Lowest rating across items 1a, 4a, 6 and 7
	Overall confidence in study findings	Longest follow up (if applicable)	Lowest rating across items 1b, 4b, 6 and 7 (N/A if 1b and 4b N/A)

* Maximum acceptable rate of differential attrition for each overall attrition rate (Source: Deke, Sama‐Miller & Hershey, 2015)

## APPENDIX F CODING TOOL

**Table:**

Category		Answer
Descriptive information	Title	Open answer
	Author citation	Open answer
	Publication Date	Open answer
	URL	Open answer
	Volume no	Open answer
	Issue no	Open answer
Geographical information	World Bank region	1. South Asia 2. Sub‐Saharan Africa 3. East Asia and Pacific 4. Europe and Central Asia 5. Latin America and Caribbean 6. Middle East and North Africa 7. North America
	Country	1. Low income 2. Lower Middle income 3. Upper Middle income 4. Conflict affected
		See relevant country list as per World Bank Region
Study design		1. Systematic reviews 2. RCT 3. Quasi‐experimental study 4. Case‐control 5. Cohort 6. Controlled trial 7. Other matching 8. Unmatched 9. No control
Population		1. Infanthood 2. Childhood 3. Adolescence 4. Adult 5. Elderly 6. Boys/men 7. Girls/women 8. Pregnant women 9. Children with disabilities 10. Ethnic minorities 11. Rural population 12. Urban population 13. LGBTQ
Type of Impairment		1. Physical impairment 2. Visual impairment 3. Mental impairment 4. Hearing impairment 5. Intellectual/learning impairment
Intervention		1. Social assistance 2. Cash transfer 3. In‐kind transfer 4. Child welfare program 5. Targeted food assistance 6. Social insurance 7. Pensions 8. Old age insurance 9. Disability pensions 10. Disability insurance 11. Health insurance 12. Unemployment insurance 13. Labour marketing 14. Active labour marketing 15. Job centres 16. Training 17. Policy to promote enterprises 18. Passive labour markets 19. Maternity benefits 20. Injury compensation 21. Sickness benefit 22. Minimum wage
Outcome		1. Social inclusion and gender equity 2. Nutrition 3. Employment 4. Education 5. Health and nutrition 6. Economic
Publication status		1. Published 2. grey literature
Region		1. East Asia and Pacific 2. Europe and Central Asia 3. North America 4. Middle East and North Africa 5. South Asia 6. Sub‐Saharan Africa 7. Latin America and Caribbean
Conflict‐affected region		1. High‐fragility 2. Medium fragility 3. Low fragility 4. Neighbours 5. Not conflict affected
Systematic review quality		1. Low confidence 2. Medium confidence 3. High confidence 4. Impact evaluation
Critical appraisal		
Funder		1. GDN 2. Bill and Melinda Gates Foundation 3. National bureau of economic research 4. World bank's research support budget 5. World bank trust funds 6. Other

## APPENDIX G SELECTION CRITERIA

**Table:**

Selection criteria	Inclusion	Exclusion
Publication status	Completed Published or unpublished	On going
Study design	The EGM will include systematic reviews and impact evaluation or effectiveness studies that used either: (a) randomized experimental design, or (b) rigorous quasi‐experimental design (d) regression discontinuity (e) propensity score matching (f) difference in difference (g) instrumental variables (h) and other matching design (i) Single subject design (j) Systematic reviews (k) Metanalysis/meta regressions (l) Natural experiments (m) Analytical observational (n) Before‐after studies (o) Time‐series	Literature reviews, non‐effectiveness studies, Case studies and qualitative studies
Population	People living in LMICs will be included. Population sub groups are: The age group is classified based on the WHO age criteria stated as follows: Infanthood (<3 years of age), Childhood (3‐11 years), Adolescence (11‐18 years), Adults (18‐60 years), elderly (above 60 years), boys/men, girls/women, pregnant women/girls, children with disability, children in low income settings, ethnic minorities.	People living in high‐income countries.
Interventions	The International Labour Organization (ILO) has defined social protection intervention as social assistance, social insurance and labour marketing facilities (ILO, 2014).	Interventions not focused on social protection. We will also exclude studies that deals general interventions which are applicable to all the people such as compulsory immunisation, minimum age for marriage etc.
	1. Social assistance (social safety nets) interventions are social/cash transfer/in‐kind transfers, child welfare programs and targeted food assistance 2. Social insurance intervention includes pensions (old age/disability), health insurance, unemployment insurance, disability insurance, maternity benefits, and provident funds 3. Intervention related to labour marketing and active labour market (job centers, job training, policies to promote small and medium sized enterprises) and passive labour market (maternity benefits, injury compensation, sickness benefits, minimum wage)	
Outcome	The outcome are social inclusion and gender equity, nutrition, employment, education, health and nutrition and economic status	None
Confidence in study findings	Low and medium confidence reviews High confidence reviews Low and medium confidence impact evaluations High confidence impact evaluations	
